# Affinity-based target deconvolution of safranal

**DOI:** 10.1186/2008-2231-21-25

**Published:** 2013-03-20

**Authors:** Hossein Hosseinzadeh, Soghra Mehri, Mohammad Mahdi Abolhassani, Mohammad Ramezani, Amirhossein Sahebkar, Khalil Abnous

**Affiliations:** 1 Pharmaceutical Research Center, Pharmacodynamics and Toxicology Department, School of Pharmacy, Mashhad University of Medical Sciences, Mashhad, Iran; 2 Pharmaceutical Research Center, Department of Medicinal Chemistry, School of Pharmacy, Mashhad University of Medical Sciences, Mashhad, Iran; 3 Pharmaceutical and Biotechnology Research Centers, School of Pharmacy, Mashhad University of Medical Sciences, Mashhad, Iran; 4 Biotechnology Research Center and School of Pharmacy, Mashhad University of Medical Sciences, Mashhad, Iran

**Keywords:** Safranal, *Crocus sativus*, Saffron, Target deconvolution, Affinity chromatography, Proteomics

## Abstract

**Background and the purpose of the study:**

Affinity-based target deconvolution is an emerging method for the identification of interactions between drugs/drug candidates and cellular proteins, and helps to predict potential activities and side effects of a given compound. In the present study, we hypothesized that a part of safranal pharmacological effects, one of the major constituent of *Crocus sativus* L., relies on its physical interaction with target proteins.

**Methods:**

Affinity chromatography solid support was prepared by covalent attachment of safranal to agarose beads. After passing tissue lysate through the column, safranal-bound proteins were isolated and separated on SDS-PAGE or two-dimensional gel electrophoresis. Proteins were identified using MALDI-TOF/TOF mass spectrometry and Mascot software.

**Results and major conclusion:**

Data showed that safranal physically binds to beta actin, cytochrome b-c1 complex sub-unit 1, trifunctional enzyme sub-unit beta and ATP synthase sub-unit alpha and beta. These interactions may explain part of safranal’s pharmacological effects. However, phenotypic and/or biological relevance of these interactions remains to be elucidated by future pharmacological studies.

## Introduction

**Figure 1:**
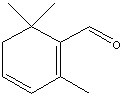
Chemical structure of safranal.

*Crocus sativus* L., commonly known as saffron, is a perennial stemless herb belonging to the Iridaceae family. The most important constituents of saffron are safranal, crocin, picrocrocin and crocetin [1]. Safranal (2,6,6-trimethyl-1,3-cyclohexadiene-1-carboxaldehyde; Figure 1) is a volatile monoterpene aldehyde that is produced by acidic hydrolysis of picrocrocin [2], and is responsible for the saffron’s unique aroma [3]. While safranal is the major component of saffron’s oil, its presence in the plant’s extract is less than 1% [4].

Saffron is a medicinal plant endowed with a plethora of biological and pharmacological activities and a long history of applications in the Islamic traditional medicine [5,6]. Safranal is believed to play a key role in the observed biological activities of saffron. Modern pharmacological studies have unveiled antioxidant [3,7,8], genoprotective [9], bronchodilatory [10], cytotoxic [2,11], antitussive [12], anticonvulsant [13,14], antinociceptive [15], neuroprotective [16], antiabsence [17], antidepressant [18], hypotensive [19], anxiolytic and hypnotic [20] effects of safranal.

Traditionally, drug discovery has been based on the observation of a phenotypic change after application of a natural or synthetic compound. Isolation and identification of molecular targets of a natural product or drug helps to elucidate its mechanism of action and side effects and also predict potential activities. This approach is called target deconvolution [21]. Finding new potential therapeutic effects or un-anticipated side effects can be considered as benefits of target deconvolution.

In the present study, we looked for the spectrum of cellular proteins that could interact with safranal. To this end, an affinity chromatography-based purification followed by two-dimensional gel electrophoresis isolation method was used. Potential targets of safranal were finally unraveled through proteomic identification.

## Material and methods

### Animals and tissue samples

Animal study was approved by the Institutional Ethics Committee. Animals were housed at temperature of 25 ± 2°C on a 12-h light/dark cycle with free access to food and water. Twelve male BALB/c mice (6 weeks old, 20–30 g weight) were sacrificed by decapitation. Liver, heart, kidney and brain of mice were washed using 0.9% normal saline solution. Tissues were immediately frozen in liquid nitrogen and stored at −80°C until use.

### Preparation of tissue extracts

Liver, heart, kidney and brain tissues (200–400 mg) were homogenized in extraction buffer [with the ratio of 1:5 (w:v)] containing 50 mM Tris (pH 7.4), 2 mM EGTA, 2 mM EDTA, 2 mM Na_3_VO_4_, 1% Triton X-100 and 10 mM 2-mercaptoethanol, with further addition of a few crystals of the protease inhibitor phenylmethylsulfonyl fluoride (PMSF) immediately before homogenization of tissue. Homogenization was performed using a Polytron Homogenizer (Kinematica, Switzerland) followed by sonication (UP100H, Hielscher) for 40 seconds. After centrifugation (Hettich Universal 320R, Germany) at 25,000 g for 10 min at 4°C, the supernatant was stored on ice. Protein contents were measured using a Bradford protein assay kit (BioRad). The same amount of proteins was used for each experiment.

### Preparation of safranal-resin conjugate

Safranal was covalently bound to matrix using PharmaLink Kit (Pierce) according to the manufacturer’s instructions. Briefly, agarose beads containing immobilized diaminodipropylamine (DADPA) side chain were equilibrated in 4 mL coupling buffer (0.1 M MES, 0.15 M NaCl, pH 4.7) and 50% ethanol. Safranal (100 mg) was dissolved in 2 mL of coupling buffer and transferred to resin slurry. Coupling reaction was started by adding 200 μL of coupling reagent (37% formaldehyde solution) to the resin/safranal mixture. Reaction mixture was incubated for 72 hrs at 50°C. Resin slurry was transferred to a column and washed 12 times each time with 2 mL of wash buffer (0.1 M Tris, pH 8.0) and 50% ethanol to remove unbound safranal. Flow-through fractions were collected and pooled. Amount of unbound safranal was calculated by measuring pooled flow through absorbance at 314.8 nm using visible spectroscopy (CECIL 9000 Series) [22].

### Affinity chromatography

Protein targets of safranal were isolated using affinity chromatography. Briefly, columns with and without (as control) bound safranal were equilibrated in binding buffer (50 mM Tris, pH 7.4, 2 mM EGTA, 2 mM EDTA, 2 mM Na_3_VO_4_, 1% Triton X-100, and 10 mM 2-mercaptoethanol). Tissue extracts were incubated with control column resin for 30 min in 4°C. After a brief centrifugation at 1,000 g for 1 min, supernatants were transferred to affinity column. After 30 min of incubation at 4°C, affinity column was washed 4 times, each time with 2 ml of binding buffer to remove unbound cellular components. Safranal target proteins were then eluted with 2 mL of 2 M NaCl in binding buffer. Elution was repeated 3 more times and fractions were pooled. Presence of proteins in fractions was tested using Bradford protein assay kit (BioRad). Pooled fractions were dialyzed using a 2,000 Da cutoff membrane in order to remove electrolytes. To concentrate target proteins, samples were freeze dried and stored at −20°C until use.

### Separation of target proteins using SDS-PAGE

Freeze dried samples from Kidney and liver were dissolved in 20 μL of homogenization buffer containing Tris 50 mM (pH: 7.4), 2 mM EDTA, 10 mM NaF, 1 mM Na_3_VO_4_, 10 mM β- glycerol-phosphate, 0.2% W/V sodium deoxycholate, 1 mM phenylmethylsulfonyl fluoride (PMSF), and complete protease inhibitor cocktail (Sigma, P8340). After addition of 20 μL of 2× SDS buffer, samples were incubated in boiling water for 5 min and then subjected to SDS-PAGE electrophoresis (BioRad). Gels were silver stained and protein bands were excised and collected in microtubes.

### Two-dimensional gel electrophoresis

Proteins were dissolved to a final concentration of 125 μg/125 μL in rehydration buffer [containing 6 M urea, 2 M thiourea, 2% CHAPS, 50 mM Dithiothreitol (DTT), 20% Bio-Lyte (BioRad)]. Non-linear immobilized pH gradient (IPG) strips (pH range: 3–10) were used to separate safranal target proteins based on their isoelectric point. Following passive rehydration at room temperature for 12 hrs, isoelectric focusing was performed using PROTEAN IEF CELL (BioRad) at 4000 V for 11 hrs. IPGs were incubated in equilibration buffer [375 mM Tris (pH 8.8), 6 M Urea, 2.5% SDS and 30% glycerol] for 20 min. IPGs were placed on top of 12% SDS-PAGE and sealed with heated agarose solution [25 mM Tris (pH 8.8), 84 mM Glycin, 0.5% agarose, 0.1% SDS and small amount of tracking dye bromophenol blue]. Electrophoresis was performed for 80 min at 120 V. Gels were silver stained and protein spots were excised and collected in microtubes.

### In-gel digestion

Gel slices were incubated in destaining buffer (50 MeOH, 5% acetic acid) at room temperature overnight. Destaining was repeated with fresh buffer for 2 more hrs. Gel slices were dehydrated in acetonitrile for 30 min and dried in vacufuge. Gels were then covered with reducing buffer (1.5 mg/mL in 100 mM ammonium bicarbonate) for 1 h. Protein alkylation was performed by incubation of gel slices in 100 μL of 10 mg/mL iodoacetamide in 100 mM ammonium bicarbonate for 30 min at room temperature. Afterwards, gel slices were washed using 0.5 mL of 100 mM ammonium bicarbonate. After dehydration (using acetonitrile) and drying (in vacufuge), 50 μL of 20 μg/mL trypsin was added to each gel slice and incubated on ice (5 min) and then overnight. Peptides were extracted in 3 steps by adding 100 μL of 100 mM ammonium bicarbonate, 100 μL extraction solution (50% acetonitrile and 5% formic acid) and 150 μL extraction solution, respectively. Samples were dried down to a final volume of 15 μL in vacufuge. Finally, samples were desalted using ZipTip® μC-18 (Millipore). Eluted samples were stored at −20°C until use.

### Mass spectrometry analysis

Mass analysis was performed at the Genome Research Centre, University of Hong Kong, using a 4800 MALDI-TOF/TOF analyzer (ABI). Data were searched against both NCBInr and SwissProt databases. Mascot software was used to analyze Mass data. Mascot search parameters were set as follow: Taxonomy: Rat, Fixed modification: Carbamidomethyl (C), Variable modification: Oxidation (M), MS/MS fragment tolerance: 0.2 Da, Precursor tolerance: 75 ppm, peptide charge: +1, monoisotopic. Proteins with a score of > 30 and confidence interval (CI) > 95% were accepted.

## Results

### Preparation of safranal-resin conjugate

**Figure 2:**
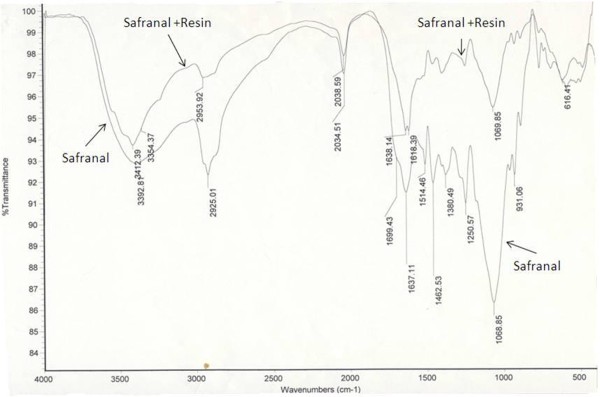
FT-IR spectrum of safranal and safranal-resin complex.

Safranal was covalently attached to DADPA chain of agarose beads using the Mannich reaction. Briefly, formaldehyde reacts with the primary amino group to produce highly reactive iminium group. This group can react with active hydrogen on safranal ring. Yield of safranal-resin conjugation was calculated to be 90%. Unbound safranal was washed away. Presence of safranal in final product was confirmed by FT-IR (Figure 2).

In the IR spectrum of safranal resin complex, the aldehyde (C=O) group of safranal was observed at 1638.14 cm^-1^, which is similar to its position in pure safranal C=O (1637.11 cm^-1^).

### Target proteins of safranal in liver

Affinity chromatography was performed to find cellular targets of safranal in different organs such as liver, heart, kidney and brain. There are two types of interactions between stationary phase and cellular proteins: specific interaction between safranal and target proteins and unspecific binding between proteins and other parts of stationary phase like agarose beads. To eliminate unspecific binding of non-target proteins, tissue extracts were incubated with control agarose beads. After a brief centrifugation, supernatant was incubated with safranal-resin stationary phase. Target proteins were eluted and subjected to two-dimensional gel electrophoresis. After in-gel digestion of protein spots, MALDI TOF/TOF mass spectrometry was used for their identification. Mass data were analyzed using Mascot software.

**Figure 3:**
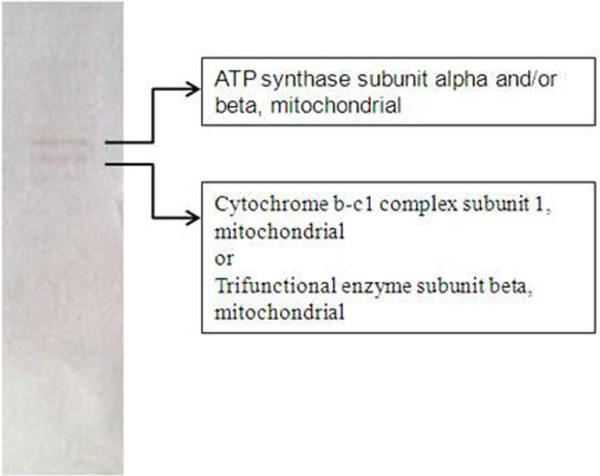
**SDS-PAGE electrophoresis of safranal targets in liver extract.** Spots were identified as cytochrome c1 complex sub-unit 1, trifunctional enzyme sub-unit beta and ATP synthase sub-unit alpha and beta.

Cytochrome b-c1 complex sub-unit 1, trifunctional enzyme sub-unit beta and ATP synthase sub-units alpha and beta were identified as safranal targets in liver (Figure 3, Table 1, Additional file 1).

**Table 1:** Molecular targets of safranal as identified by MALDI-TOF/TOF mass spectrometry and Mascot

	**Protein name**	**Protein score**	**Protein score C.I. %**	**MW/pI**
1	ATP synthase sub-unit alpha	**485**	**100**	**56 KDa/5.19**
2	ATP synthase sub-unit beta	**485**	**100**	**56 KDa/5.19**
3	Beta-actin-like protein 2	**215**	**100**	**42 KDa/5.3**
4	Cytochrome b-c1 complex sub-unit 1	**265**	**100**	**53 KDa/5.75**
5	Trifunctional enzyme sub-unit beta	**258**	**100**	**51 KDa/4.82**

### Targets of safranal in kidney, heart and brain

**Figure 4:**
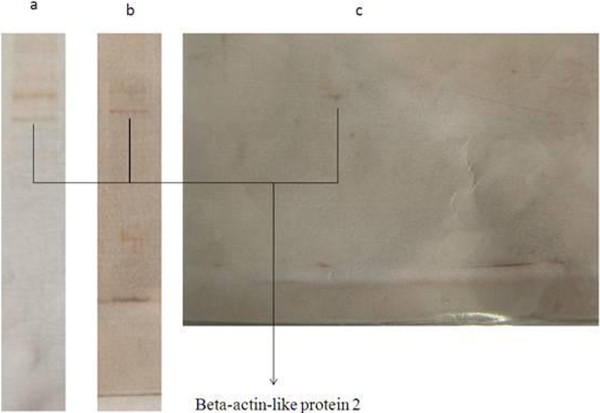
**SDS-PAGE and two-dimensional gel electrophoresis of safranal targets in heart (a), kidney (b) and brain(c) extract.** Spots were identified as Beta-actin-like protein 2.

Beta-actin-like protein 2 was identified as cellular targets of safranal in kidney, heart and brain (Figure 4, Table 1, Additional file 1).

## Discussion

Drugs are normally discovered based on their ability to show a certain desired biological outcome. The conventional approach for drug discovery from natural resources mainly starts with the pharmacological screening of crude extracts followed by sequential fractionation and finally isolation and purification of bioactive phytochemicals. During recent years, the advent of high-throughput mass spectrometry-based techniques has enabled the scientists to unveil molecular targets of small molecules of either natural or synthetic entity. Such a target deconvolution strategy not only provides an invaluable solution for exploring the molecular mechanisms behind the observed phenotypic effects of a drug/drug candidate (retrospective approach), but is also a useful tool for predicting the potential biological and pharmacological activities of any natural or lead compound prior to the start of pharmacological studies. Having known the molecular targets, prediction of plausible adverse events is also possible due to the well-documented roles of numerous proteins in the pathogenesis of certain disorders [21].

Affinity-based target deconvolution methods always carry the risk of identifying interactions with proteins that have no pharmacological relevance (false positives), despite being targets of the compound. Activity or phenotype based assays are essential to discriminate between positive and false-positive interactions and confirm functional effects [21].

In the present study, the fact that physical interaction is a prerequisite for functional effects was used to affinity purify target proteins of safranal. Our data showed that safranal binds to beta actin, cytochrome b-c1 complex sub-unit 1, trifunctional enzyme sub-unit beta, and ATP synthase sub-units alpha and beta.

Beta actin like protein 2 was identified as one of the safranal protein targets. Actin filaments help in maintaining cell morphology and functions such as adhesion, motility, exocytosis, endocytosis and cell division. Natural products like cytochalasin and jasklapinolide that interact with actin polymerization have cytotoxic effects [23]. In previous studies, saffron and safranal were shown to possess cytotoxic activities and inhibit the growth of human cancer cells. In light of the present findings, depolymerization of actin filaments by safranal could explain part of the observed cytotoxic effects of safranal [24,25]. However, a possible contribution of antioxidant properties to the cytotoxicity of safranal needs further investigation due to the controversies over the consequence of blunting ROS on the fate of cancerous cell [26,27]. Reported cytotoxic effects of antioxidants, including safranal, have been mainly based on *in vitro* cell line studies, for which serious arguments have been raised [28]. In addition, it has been proposed that antioxidant therapy may deplete cellular reservoir of hydrogen peroxide, thereby inhibiting subsequent inhibition of neovascularization and metabolism [29].

Aside from the aforementioned effects, safranal also interacts with cytochrome b-c1. The most conserved role of these cytochromes is in the electron transport chain powering oxidative phosphorylation. Moreover, cytochrome *c* release into the cytosol is particularly associated with activation of the intrinsic apoptotic pathway [30].

The mitochondrial trifunctional protein (MTP) is a heterotrimeric protein that consists of four α-sub-units and four β-sub-units and catalyzes the mitochondrial β-oxidation of long-chain fatty acids. Safranal may alter mitochondrial fatty acid oxidation by binding to this enzyme [31].

ATP synthase is a key enzyme of mitochondrial energy conversion [32]. Ahmad and Laughlin [33] discussed that dietary polyphenols and amphibian antimicrobial/antitumor peptides inhibit ATP synthase. Inhibition of ATP synthase may cause energy deprivation and increase ROS production. High ROS content induces cellular necrosis and/or apoptosis [32]. Our experiment showed that safranal may physically interact with this enzyme.

Although physical interaction with cellular proteins is a prerequisite for pharmacological effects of drugs in many instances, biological relevance of such interactions remain to be elucidated by appropriately designed experimental investigations. The necessity of conducting such investigations would be more evident when taking into account the fact that many drug-target interactions do not translate into a significant alteration in the protein function and thereby any considerable pharmacological or clinical effect.

## Conclusion

Evidence from the present study suggested that beta actin, cytochrome b-c1 complex sub-unit 1, trifunctional enzyme sub-unit beta and ATP synthase sub-unit alpha and beta could be regarded as potential cellular targets of safranal. Activity- or phenotype-based assays are essential to elucidate the inhibitory or stimulatory effects of safranal on its targets.

## Competing interests

The authors report no declarations of interest.

## Authors’ contributions

KA, MR and HH conceived the study and designed the experiments. SM and MMA performed the experimental work. KA, MMA and AS were involved in data interpretation and drafting the manuscript. All authors read and approved the final manuscript.

## Supplementary Material

Additional file 1 Results from Mascot search are available as supporting information. Click here for file
