# Supplementary material for: Affinity-based target deconvolution of safranal
Source: Daru. 2013 Mar 20;21(1):25. doi: 10.1186/2008-2231-21-25 (PMC3614472; doi:10.1186/2008-2231-21-25)
Supplement: Additional file 1 — Results from Mascot search are available as supporting information. [file 2008-2231-21-25-S1.pdf]

Results from MASCOT search

1. Beta-actin-like protein 2

Analysis Information

Report Type	Protein-Peptide Summary by Spot	Analysis Type	Combined (MS+MS/MS)
Sample Set Name	2011-05-04 [001300010097-2011-03-18-1]	Database	SwissProt
Analysis Name	Khalil-Medicinal Chemistry[Mus Swissprot]	Creation Date	05/04/2011 13:12:29
Reported By	05/04/2011 17:22:24 - admin	Last Modified	05/04/2011 13:18:54

MS Acq. : Proc. Methods	(Unspecified) : (Unspecified)
Interpretation Method	(Unspecified)

Gel Idx/Pos	325/N3	Instr./Gel Origin	Ak115/2011-03-18-1	Process Status	Analysis Succeeded
Plate [#] Name	[1] 001300010097	Instrument Sample Name		Spectra	6

Rank	Protein Name	Accession No.	Protein Score	Protein Score C. I. %	Total Ion Score	Total Ion C. I. %	Protein MW	Protein PI	Spot Name	Pep. Count	User name	Department
1	RecName: Full=Beta-actin-like protein 2; AltName: Full=Kappa-actin	gi|81895966|sp|Q8BFZ3.1|ACTBL_MOUSE	224	100	194	100	42319.1	5.3	A-K-1	6	Khalil	Medicinal Chemistry

Peptide Information

Calc. Mass	Obsrv. Mass	± da	± ppm	Start Seq.	End Seq.	Sequence	Ion Score	C. I. %	Modification	Rank	Result Type
923.5672	923.5939	0.0267	29	330	337	IIAPPERK					Mascot
1036.6514	1036.6608	0.0094	9	328	336	IKIIAPPER					Mascot
1232.6569	1232.6783	0.0214	17	30	40	AVFPSMVGRPR			Oxidation (M)[6]		Mascot
1639.8359	1639.9365	0.1006	61	179	192	LDLAGRDLTDYLMK			Oxidation (M)[13]		Mascot
1790.892	1790.9762	0.0842	47	240	255	SYELPDGQVITIGNER	131	100			Mascot
1790.892	1790.9762	0.0842	47	240	255	SYELPDGQVITIGNER					Mascot
1954.0645	1954.1536	0.0891	46	97	114	VAPDEHPILLTEAPLNPK	63	99.999			Mascot
1954.0645	1954.1536	0.0891	46	97	114	VAPDEHPILLTEAPLNPK					Mascot

2. Cytochrome b-c1 complex subunit 1

Analysis Information

Report Type	Protein-Peptide Summary by Spot	Analysis Type	Combined (MS+MS/MS)
Sample Set Name	2011-05-04 [001300010097-2011-03-18-1]	Database	SwissProt
Analysis Name	Khalil-Medicinal Chemistry[Mus Swissprot]	Creation Date	05/04/2011 13:12:29
Reported By	05/04/2011 17:22:25 - admin	Last Modified	05/04/2011 13:18:54

MS Acq. : Proc. Methods	(Unspecified) : (Unspecified)
Interpretation Method	(Unspecified)

Gel Idx/Pos	327/N5	Instr./Gel Origin	Ak115/2011-03-18-1	Process Status	Analysis Succeeded
Plate [#] Name	[1] 001300010097	Instrument Sample Name		Spectra	6

Rank	Protein Name	Accession No.	Protein Score	Protein Score C. I. %	Total Ion Score	Total Ion C. I. %	Protein MW	Protein PI	Spot Name	Pep. Count	User name	Department
1	RecName: Full=Cytochrome b-c1 complex subunit 1, mitochondrial; AltName: Full=Complex III subunit 1	gi|14548301|sp|Q9CZ13.1|QCR1_MOUSE	265	100	196	100	53419.7	5.75	A-L-1	11	Khalil	Medicinal Chemistry

Peptide Information

Calc. Mass	Obsrv. Mass	± da	± ppm	Start Seq.	End Seq.	Sequence	Ion Score	C. I. %	Modification	Rank	Result Type
912.4396	912.4569	0.0173	19	473	479	SGMFWLR			Oxidation (M)[3]		Mascot
1059.5081	1059.5363	0.0282	27	473	480	SGMFWLRF			Oxidation (M)[3]		Mascot
1100.5735	1100.6115	0.038	35	424	432	IPLAEWESR					Mascot
1110.5426	1110.5802	0.0376	34	214	222	TDLTDYLNR					Mascot
1202.6198	1202.6586	0.0388	32	433	442	IQEVDAQMLR					Mascot
1218.6147	1218.6503	0.0356	29	433	442	IQEVDAQMLR			Oxidation (M)[8]		Mascot
1256.6746	1256.7067	0.0321	26	423	432	RIPLAEWESR	41	99.719			Mascot
1256.6746	1256.7067	0.0321	26	423	432	RIPLAEWESR					Mascot
1538.7479	1538.8057	0.0578	38	379	392	LCTSATESEVTRGK			Carbamidomethyl (C)[2]		Mascot
1605.7577	1605.7983	0.0406	25	256	269	VYEEDAVPGLTPCR	71	100	Carbamidomethyl (C)[13]		Mascot
1605.7577	1605.7983	0.0406	25	256	269	VYEEDAVPGLTPCR			Carbamidomethyl (C)[13]		Mascot
1646.8132	1646.8577	0.0445	27	112	126	EVESIGAHLNAYSTR					Mascot
2053.9971	2054.0491	0.052	25	397	415	NALVSHLDGTTPVCEDIGR	84	100	Carbamidomethyl (C)[14]		Mascot
2053.9971	2054.0491	0.052	25	397	415	NALVSHLDGTTPVCEDIGR			Carbamidomethyl (C)[14]		Mascot
2602.3259	2602.3831	0.0572	22	112	134	EVESIGAHLNAYSTREHTAYLIK					Mascot

3. ATP synthase subunit alpha

Analysis Information

Report Type	Protein-Peptide Summary by Spot	Analysis Type	Combined (MS+MS/MS)
Sample Set Name	2011-05-04 [001300010097-2011-03-18-1]	Database	SwissProt
Analysis Name	Khalil-Medicinal Chemistry[Mus Swissprot]	Creation Date	05/04/2011 13:12:29
Reported By	05/04/2011 17:22:26 - admin	Last Modified	05/04/2011 13:18:54

MS Acq. : Proc. Methods	(Unspecified) : (Unspecified)
Interpretation Method	(Unspecified)

Gel Idx/Pos	328/N6	Instr./Gel Origin	Ak115/2011-03-18-1	Process Status	Analysis Succeeded
Plate [#] Name	[1] 001300010097	Instrument Sample Name		Spectra	6

Rank	Protein Name	Accession No.	Protein Score	Protein Score C. I. %	Total Ion Score	Total Ion C. I. %	Protein MW	Protein PI	Spot Name	Pep. Count	User name	Department
2	RecName: Full=ATP synthase subunit alpha, mitochondrial; Flags: Precursor	gi|416677|sp|Q03265.1|ATPA_MOUSE	139	100	97	100	59829.6	9.22	A-L-2	10	Khalil	Medicinal Chemistry

Peptide Information

Calc. Mass	Obsrv. Mass	± da	± ppm	Start Seq.	End Seq.	Sequence	Ion Score	C. I. %	Modification	Rank	Result Type
1000.6036	1000.6279	0.0243	24	124	132	LIKEGDVVK					Mascot
1026.5942	1026.6487	0.0545	53	195	204	AVDSLVPIGR					Mascot
1120.7201	1120.7817	0.0616	55	172	182	VGLKAPGIIPR					Mascot
1229.6848	1229.7469	0.0621	51	208	218	ELIIGDRQTGK					Mascot
1358.7461	1358.8114	0.0653	48	183	194	ISVREPMQTGIK					Mascot
1374.741	1374.8071	0.0661	48	183	194	ISVREPMQTGIK			Oxidation (M)[7]		Mascot
1438.8489	1438.9028	0.0539	37	403	416	GIRPAINVGLSVSR					Mascot
1439.8264	1439.8893	0.0629	44	323	334	QMSLLLRRPPGR			Oxidation (M)[2]		Mascot
1553.7383	1553.8164	0.0781	50	335	347	EAYPGDVFYLHSR	97	100			Mascot
1553.7383	1553.8164	0.0781	50	335	347	EAYPGDVFYLHSR					Mascot
1624.8904	1624.962	0.0716	44	134	149	TGAIVDVPVGEELLGR					Mascot
1780.9916	1781.047	0.0554	31	133	149	RTGAIVDVPVGEELLGR					Mascot

4. ATP synthase subunit beta

Analysis Information

Report Type	Protein-Peptide Summary by Spot	Analysis Type	Combined (MS+MS/MS)
Sample Set Name	2011-05-04 [001300010097-2011-03-18-1]	Database	SwissProt
Analysis Name	Khalil-Medicinal Chemistry[Mus Swissprot]	Creation Date	05/04/2011 13:12:29
Reported By	05/04/2011 17:22:26 - admin	Last Modified	05/04/2011 13:18:54

MS Acq. : Proc. Methods	(Unspecified) : (Unspecified)
Interpretation Method	(Unspecified)

Gel Idx/Pos	328/N6	Instr./Gel Origin	Ak115/2011-03-18-1	Process Status	Analysis Succeeded
Plate [#] Name	[1] 001300010097	Instrument Sample Name		Spectra	6

Rank	Protein Name	Accession No.	Protein Score	Protein Score C. I. %	Total Ion Score	Total Ion C. I. %	Protein MW	Protein PI	Spot Name	Pep. Count	User name	Department
1	RecName: Full=ATP synthase subunit beta, mitochondrial; Flags: Precursor	gi|20455479|sp|P56480.2|ATPB_MOUSE	485	100	367	100	56265.5	5.19	A-L-2	15	Khalil	Medicinal Chemistry

Peptide Information

Calc. Mass	Obsrv. Mass	± da	± ppm	Start Seq.	End Seq.	Sequence	Ion Score	C. I. %	Modification	Rank	Result Type
1262.6409	1262.7026	0.0617	49	110	121	TIAMDGTEGLVR					Mascot
1385.7094	1385.7786	0.0692	50	144	155	IMNVIGEPIDER					Mascot
1401.7042	1401.7753	0.0711	51	144	155	IMNVIGEPIDER			Oxidation (M)[2]		Mascot
1406.681	1406.7507	0.0697	50	226	239	AHGGYSVFAGVGER	127	100			Mascot
1406.681	1406.7507	0.0697	50	226	239	AHGGYSVFAGVGER					Mascot
1435.7539	1435.8242	0.0703	49	311	324	FTQAGSEVSALLGR					Mascot
1439.7893	1439.8893	0.1	69	282	294	VALTGLTVAEYFR					Mascot
1601.8104	1601.886	0.0756	47	265	279	VALVYGQMNEPPGAR	55	99.992			Mascot
1601.8104	1601.886	0.0756	47	265	279	VALVYGQMNEPPGAR					Mascot
1617.8053	1617.8818	0.0765	47	265	279	VALVYGQMNEPPGAR			Oxidation (M)[8]		Mascot
1650.9174	1650.9941	0.0767	46	95	109	LVLEVAQHLGESTVR	78	100			Mascot
1650.9174	1650.9941	0.0767	46	95	109	LVLEVAQHLGESTVR					Mascot
1780.9625	1781.047	0.0845	47	144	159	IMNVIGEPIDERGPIK					Mascot
1796.9575	1797.0184	0.0609	34	144	159	IMNVIGEPIDERGPIK			Oxidation (M)[2]		Mascot
1842.8802	1842.9714	0.0912	49	407	422	IMDPNIVGNEHYDVAR					Mascot
1858.8752	1858.9777	0.1025	55	407	422	IMDPNIVGNEHYDVAR			Oxidation (M)[2]		Mascot
1919.0959	1919.1879	0.092	48	125	143	VLDSGAPIKIPVGPETLG	107	100			Mascot

5. Trifunctional enzyme subunit beta

Analysis Information

Report Type	Protein-Peptide Summary by Spot	Analysis Type	Combined (MS+MS/MS)
Sample Set Name	2011-05-04 [001300010097-2011-03-18-1]	Database	SwissProt
Analysis Name	Khalil-Medicinal Chemistry[Mus Swissprot]	Creation Date	05/04/2011 13:12:29
Reported By	05/04/2011 17:22:26 - admin	Last Modified	05/04/2011 13:18:54

MS Acq. : Proc. Methods	(Unspecified) : (Unspecified)
Interpretation Method	(Unspecified)

Gel Idx/Pos	328/N6	Instr./Gel Origin	Ak115/2011-03-18-1	Process Status	Analysis Succeeded
Plate [#] Name	[1] 001300010097	Instrument Sample Name		Spectra	6

Rank	Protein Name	Accession No.	Protein Score	Protein Score C. I. %	Total Ion Score	Total Ion C. I. %	Protein MW	Protein PI	Spot Name	Pep. Count	User name	Department
2	RecName: Full=Trifunctional enzyme subunit beta, mitochondrial; AltName: Full=TP-beta; Includes: Re	gi|51316075|sp|Q99JY0.1|ECHB_MO	91	99.999	27	94.125	51638.5	9.43	A-L-1	11	Khalil	Medicinal Chemistry

Analysis Information

Report Type	Protein-Peptide Summary by Spot	Analysis Type	Combined (MS+MS/MS)
Sample Set Name	2011-05-04 [001300010097-2011-03-18-1]	Database	SwissProt
Analysis Name	Khalil-Medicinal Chemistry[Mus Swissprot]	Creation Date	05/04/2011 13:12:29
Reported By	05/04/2011 17:22:25 - admin	Last Modified	05/04/2011 13:18:54

MS Acq. : Proc. Methods	(Unspecified) : (Unspecified)
Interpretation Method	(Unspecified)

USE

Peptide Information

Calc. Mass	Obsrv. Mass	± da	± ppm	Start Seq.	End Seq.	Sequence	Ion Score	C. I. %	Modification	Rank	Result Type
905.5203	905.5383	0.018	20	231	239	LAAAFAVSR					Mascot
937.5577	937.5787	0.021	22	83	91	AALSGLLHR	27	94.125			Mascot
937.5577	937.5787	0.021	22	83	91	AALSGLLHR					Mascot
984.5836	984.6089	0.0253	26	54	62	NIVVVEGVR					Mascot
1055.6095	1055.5413	-0.0682	-65	408	417	TKVGSPPLEK					Mascot
1154.5146	1154.5502	0.0356	31	240	248	MEQDEYALR					Mascot
1170.5095	1170.5463	0.0368	31	240	248	MEQDEYALR			Oxidation (M)[1]		Mascot
1545.7689	1545.8134	0.0445	29	279	292	DNGIRPSSLEQMAK					Mascot
1561.7639	1561.8085	0.0446	29	279	292	DNGIRPSSLEQMAK			Oxidation (M)[12]		Mascot
1683.8701	1683.9056	0.0355	21	255	269	KAQDEGHLSDIVPFK					Mascot
1691.6941	1691.743	0.0489	29	393	406	AMDSDWFAQNYMGR					Mascot
1714.8799	1714.9293	0.0494	29	336	349	AYLRDFIYVSQDPK					Mascot
1777.8538	1777.902	0.0482	27	240	254	MEQDEYALRSHSLAK					Mascot
1868.0786	1867.9825	-0.0961	-51	46	62	TLAKPNMKNIVVVEGVR					Mascot
